# The phylogeography of *Myotis* bat-associated rabies viruses across Canada

**DOI:** 10.1371/journal.pntd.0005541

**Published:** 2017-05-19

**Authors:** Susan Nadin-Davis, Noor Alnabelseya, M. Kimberly Knowles

**Affiliations:** 1 Animal Health Microbiology Research, Ottawa Laboratory Fallowfield, Canadian Food Inspection Agency, Ottawa, ON, Canada; 2 Department of Biochemistry, Microbiology and Immunology, University of Ottawa, Ottawa, ON, Canada; 3 Centre of Expertise for Rabies, Ottawa Laboratory Fallowfield, Canadian Food Inspection Agency, Ottawa, ON, Canada; Wistar Institute, UNITED STATES

## Abstract

As rabies in carnivores is increasingly controlled throughout much of the Americas, bats are emerging as a significant source of rabies virus infection of humans and domestic animals. Knowledge of the bat species that maintain rabies is a crucial first step in reducing this public health problem. In North America, several bat species are known to be rabies virus reservoirs but the role of bats of the *Myotis* genus has been unclear due to the scarcity of laboratory confirmed cases and the challenges encountered in species identification of poorly preserved diagnostic submissions by morphological traits alone. This study has employed a collection of rabid bat specimens collected across Canada over a 25 year period to clearly define the role of particular *Myotis* species as rabies virus reservoirs. The virus was characterised by partial genome sequencing and host genetic barcoding, used to confirm species assignment of specimens, proved crucial to the identification of certain bat species as disease reservoirs. Several variants were associated with *Myotis* species limited in their Canadian range to the westernmost province of British Columbia while others were harboured by *Myotis* species that circulate across much of eastern and central Canada. All of these *Myotis*-associated viral variants, except for one, clustered as a monophyletic MYCAN clade, which has emerged from a lineage more broadly distributed across North America; in contrast one distinct variant, associated with the long-legged bat in Canada, represents a relatively recent host jump from a big brown bat reservoir. Together with evidence from South America, these findings demonstrate that rabies virus has emerged in the *Myotis* genus independently on multiple occasions and highlights the potential for emergence of new viral-host associations within this genus.

## Author summary

Reducing the public health burden of rabies is most effectively achieved by elimination of the disease from its hosts. While the role of dogs and many wild carnivore species in maintaining and transmitting rabies virus is well established, our understanding of the role of many bat species in this regard is still incomplete. Several North American bat species are known to be rabies virus reservoirs, but the role of bats of the *Myotis* genus has been unclear due to the very limited number of laboratory confirmed rabies cases detected in these bats and the challenge in assigning species to poorly preserved bat carcasses. Our study utilised a collection of rabid bats collected across Canada over 25 years to address this issue. Genetic barcoding was used to identify the specimens to species and the virus was characterised by partial genome sequencing. Host barcoding proved to be crucial for correct species assignment to many specimens and allowed the identification of certain *Myotis* species as reservoirs for several genetically distinct variants of the rabies virus. The geographical distribution of these viral variants, and its correlation with the ecological and taxonomic properties of the hosts, are described. While most of these variants belong to a single rabies virus lineage, one variant associated with the long-legged bat appears to have emerged relatively recently following transmission from a big brown bat reservoir. Consistent with this observation, comparison of the rabies viruses associated with *Myotis* bats in North and South America clearly shows that rabies has emerged in members of this genus on several separate occasions.

## Introduction

With the emergence of several recently identified viruses, including Hendra, Nipah and SARS viruses, members of the order Chiroptera have assumed significant importance as the presumptive reservoirs of many zoonotic diseases [1]. Most of the 14 recognised species of the *Lyssavirus* genus, the etiological agents of rabies, are associated with various bat species in many parts of the world [2]. Moreover genetically distinct members of this genus continue to be identified in chiropteran hosts [3,4] while the known range of previously identified species such as Irkut virus expands [5]. In the Americas, many bat species exhibiting a variety of feeding habits, especially those with haematophagous and insectivorous diets, have been identified as reservoirs for classical rabies virus (RABV); indeed RABV is the only known circulating lyssavirus species in the Americas and only on this continent do bats act as RABV hosts. Throughout North and South America, members of three chiropteran families, the Phyllostomidae, Molossidae and the Vespertilionidae, are the most important RABV reservoirs with genetically distinct viral variants associated with several bat species [6–11]. Since the first isolation of RABV from North American bats in the 1950s [2], increased surveillance currently identifies hundreds of bat rabies cases annually across the United States and Canada [12–14].

Compared to the rest of the Americas, climatic and habitat limitations have significantly restricted the number of bat species indigenous to Canada. Only 18 species are considered indigenous and most are restricted in range to southern regions of the country [15]. The species most commonly reported as rabid in Canada is the big brown bat, (*Eptesicus fuscus*); its proclivity to roost in human dwellings, sometimes in colonies of some size, results in relatively frequent contact with humans and may possibly contribute to its perceived importance as a RABV reservoir. Five distinct viral variants (BB1 to BB5) are known to circulate within this species [16]. Less frequently reported species include hoary (*Lasiurus cinereus*), red (*L*. *borealis*) and silver-haired (*Lasionycteris noctivagans*) bats, each of which harbours its own distinctive RABV variant [17], while the rarely reported tri-coloured bat (*Perimyotis subflavus*), previously referred to as the eastern pipistrelle (*Pipistrellus subflavus*), harbours a variant closely related to that of the silver-haired bat both in Canada and the United States [18]. The relatively low submission rates for these latter solitary, migratory species may be due, at least in part, to their non-conspicuous roosting habits that result in less frequent human contact and a reduced level of passive surveillance [14]. In contrast, the role of bats of the *Myotis* genus has been more difficult to define. The gregarious little brown bat (*M*. *lucifugus*) can form colonies of some size but submissions are only rarely rabid so their role as disease reservoirs remained unclear for some time.

Antigenic and genetic analysis of a small group of heterogeneous RABVs associated with Canadian *Myotis* bats identified a variant designated as the MYCAN type [17]. Unfortunately, the small sample number and the challenges encountered in the accurate species identification of members of the genus precluded any conclusions regarding the reservoir role of particular *Myotis* species.

The advent of genetic barcoding to facilitate species identification [19] has provided an important tool for improved identification of bats. Speciation based only on morphological keys can be challenging and often results in species misidentification especially for poorly preserved specimens originating from an area harbouring multiple species. Such is the case for the southern region of the province of British Columbia, where several *Myotis* species exhibiting only subtle morphological differences overlap in range [20]. Incorporation of barcoding into a study of bat-associated RABVs of the United States defined 18 distinct viral lineages and identified several additional bat species that appear to function as maintenance hosts [11]. While some of these species are not native to Canada, several *Myotis* specimens were included and the study identified the Yuma bat (*M*. *yumanensis)* as a RABV reservoir for the first time.

In this study, phylogenetic characterization of RABVs, associated with a collection of rabid barcoded bats of the *Myotis* genus recovered from across Canada over a 25 year period, defines several distinct localised viral variants that are associated with particular species. Despite some pronounced east-west segregation, all of the *Myotis*-associated viral variants, except for one, group within a strongly supported monophyletic clade. Comparison with many viral isolates collected from bats in the United States confirms that many of these variants circulate south of the border. Phylogenetic examination of all chiropteran RABV variants of the Americas clearly demonstrates that *Myotis-*associated RABV lineages have emerged independently on multiple occasions throughout the continent.

## Methods

### Ethics statement

All biological specimens employed in this study had been submitted post-mortem for rabies diagnosis by the Rabies Laboratories of the Canadian Food Inspection Agency. The authors had full access to this collection. The procedures used to propagate virus in mice were approved by the institutional Animal Care Committee at the CFIA’s Ottawa Laboratory Fallowfield and followed the guidelines set by the Canadian Council on Animal Care.

### Bat specimens and rabies diagnosis

All bat specimens employed in this study were collected through a passive surveillance process and submitted for rabies diagnostic testing to one of two Canadian Food Inspection Agency (CFIA) laboratories located in Ottawa, Ontario, and Lethbridge, Alberta. Submissions were first evaluated morphologically to determine species and rabies diagnosis was then performed on brain smears using the standard direct fluorescent antibody test (FAT), as described [21]. Carcasses and brain tissue from rabid specimens were placed in long-term storage at -80°C. In some cases, virus was propagated by passage in suckling mice [22].

A listing of all available specimens collected between the early 1990s until 2015 is provided in S1 Table. A small number of poorly preserved specimens simply identified as a bat were included to maximise the total numbers of *Myotis* samples included in the study. Specimens are designated as follows: a two digit number to indicate year of recovery, a two letter code representing province of origin, followed by a four or five digit submission number and finally a three letter suffix designating the bat species according to either morphological or barcoding analysis, as indicated in the figure legends. The provincial codes are: AB, Alberta; BC, British Columbia; MB, Manitoba; NB, New Brunswick; NL, Newfoundland and Labrador; NS, Nova Scotia; ON, Ontario; PE, Prince Edward Island; QC, Quebec; SK, Saskatchewan. Bat species abbreviations are defined in Table 1 and the supplementary tables.

**Table 1:** Summary of host distribution of MYCAN viral variants.

Sub-clade	Variant	Host reservoir species	Summary of specimens infected with variant	Total number of specimens
MYO-I	CL	California bat (CLB)	15 CLB, 2 LBB (1 unconfirmed to species), 2 KEB, 1 YUB, 1 Human	21
	LE-1	Western long-eared bat (LEB)	18 LEB (1 unconfirmed), 1 CLB, 1 LLB, 2 KEB, 1 YUB	23
	LE-2	Western long-eared bat (LEB)	6 LEB, 1 YUB	7
	KE	Keen’s bat (KEB)	3 KEB, 1 WEB	4
	YU	Yuma bat (YUB)	13 YUB, 5 LBB, 2 KEB	20
MYO-II	LB-EAST	Little brown bat (LBB)	18 LBB	18
	NL-EAST	Northern long-eared bat (NLB)	11 NLB (2 unconfirmed), 5 LBB (2 unconfirmed), 1 ESB, 1 red fox	18
	MY-EAST	Unknown	1 LBB	1
	MY-CENTRAL	Unknown	2 NLB, 1 LBB, 1 LEB,1 dog, 1 cat	6
	MY-WEST-1	Unknown	2 CLB, 1 LBB, 1 WSB	4
	MY-WEST-2	Unknown	2 LEB	2

Abbreviations for bat hosts which are not reservoir hosts for the MYCAN lineage are: ESB, Eastern small-footed bat; LLB, Long-legged bat; WEB, Townsend’s big-eared bat; WSB, Western small-footed bat.

### Bat barcoding

To confirm species identification, genetic barcoding that targeted a portion of the cytochrome oxidase subunit 1 (COI) gene was performed. The DNA was extracted from tissue (lung or skin that included several hair follicles) recovered from each carcass, and employed as template for COI amplification as previously described [20] or, in some cases, a universal primer pair suitable for COI PCR of all indigenous Canadian bat species was employed [23]. For some specimens for which a carcass was no longer available, total RNA recovered from original bat brain tissue was used as template for a reverse transcriptase-PCR (RT-PCR) in which cDNA was primed with the reverse sense primer. Amplicons were purified using a Wizard PCR purification kit (Promega) and sequenced on both strands using the PCR primers with a BigDye Terminator v3.1 cycle sequencing kit (Applied Biosystems). Reactions were analyzed on a 3500xl genetic analyzer (Applied Biosystems) and consensus sequences, compiled using the Variant Reporter v1 software, were exported in fasta format.

The final dataset comprised 516 bp sequences from the 5’ terminal region of the COI gene. Specimens were analysed phylogenetically by comparison to a set of 17 vouchered reference sequences, as well as selected samples examined previously (See complete list in S2 Table); additional confirmation employed comparison of selected sequences with those held in the Barcode of life database (BOLD) accessed at http://www.boldsystems.org/index.php/Login/page.

### RABV characterization

Total RNA was recovered from either original bat brain or RABV-infected mouse brain using TRIzol reagent as recommended (Life Technologies). Amplification of the RABV N gene was achieved by a reverse transcription using the forward sense primer, RV-For2 (5’- GTACGCTTAACAACAARAYCARAGAA-3’) that targets the 3’ terminal sequence of the RABV genome, followed by PCR using primers RV-For2 and RabN5 essentially as described [24] to generate an amplicon of 1536 bp. Sequencing of the complete N gene (1353 bp) was performed using primers targeting internal sequence as described for the COI amplicon.

### Phylogenetic analysis

MEGA v5.1 software [25] was used to generate nucleotide sequence alignments and to perform phylogenetic analyses. Trees of COI sequence data were generated using the neighbor joining (NJ) method in which evolutionary distances were computed using the Maximum Composite Likelihood method. The RABV sequences were analysed using Maximum likelihood (ML) methods employing the T92+G+I substitution model best supported by these datasets. Datasets comprising viral sequences recovered continent-wide were analysed either by the ML method or by a coalescent approach employing the BEAST v1.75 package [26]. Log likelihood values identified the most highly supported runs as those employing the HKY+G+I model, in which distinct rates of change for codon positions 1and 2 and codon position 3 were supported, with a constant population size and relaxed molecular clock. Duplicate runs of 40 million generations, conducted with sampling every 4,000 generations with discard of the first 10% as burn-in, reached convergence and yielded effective samples sizes > 200 for all parameters. These combined runs were used with LogCombiner to generate maximum clade credibility trees.

### RABV variant mapping

Maps illustrating the distribution of the RABV variants identified in the study were produced using the ArcView GIS v.10 software. Data on ecoprovince distribution for British Columbia were obtained from the Ministry of Environment, BC, at https://catalogue.data.gov.bc.ca/dataset/ecoprovinces-ecoregion-ecosystem-classification-of-britishcolumbia under an open government license_._

## Results

### Bat barcoding

**Fig 1:**
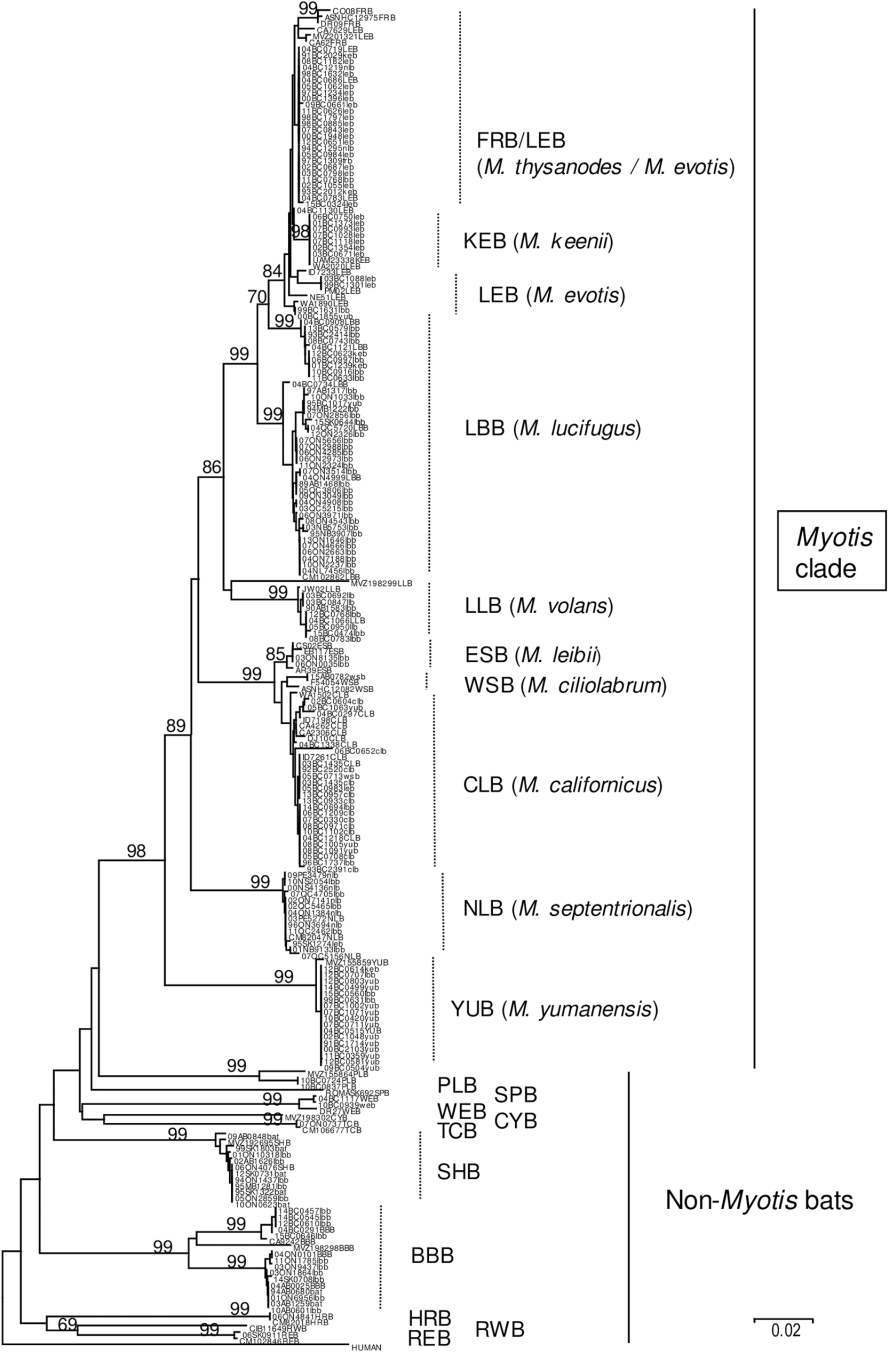
Barcoding of bat specimens. A NJ tree was generated using 216 COI sequences of 516 bp length. The sample name for 149 sequences generated during this study includes the bat species as identified morphologically in lower case while 66 reference sequences are identified by their species code in upper case (see S1 and S2 Tables); a human sequence is included as outlier. Bootstrap (bt) values for all major nodes are indicated. The species assigned to each clade is indicated to the right of the tree.

Of 161 bat specimens included in this study (see list in S1 Table), 149 were subjected to barcoding; lack of appropriate material precluded such analysis for the remaining 12 specimens. A NJ tree generated from these 149 COI sequences, together with 66 reference barcodes summarised in S2 Table and a human sequence as outgroup (Fig 1), illustrates that most of these samples can be identified to species according to their clustering patterns. Despite efforts to focus on *Myotis* species, several specimens represented species of other genera. Thus, the dataset comprised 10 silver-haired bats, of which five had been included as specimens unidentified to species due to poor sample preservation, while the remaining five had been identified originally as little brown bats. In addition, of 12 specimens that were barcoded as big brown bats, two had been included as unidentified species while the rest were also originally assigned as little brown bats. One specimen tentatively identified as a western big-eared bat was confirmed in this study.

Identification of bats within the *Myotis* genus was less clear-cut since not all species of this genus form monophyletic clades by COI sequence analysis as has been noted previously [11]. Single clades identified Yuma, northern long-eared and long-legged bats with high confidence (bt of 99 in each case). Another clade defined the *Myotis californicus* complex (bt of 99) comprising three distinct groupings: an outlying branch comprising three reference specimens (AR39ESB, CS02ESB and EB117ESB) of the eastern small-footed bat, as well as two isolates from southwestern Ontario (03ON8135lbb and 06ON0035lbb), originally identified as little brown bats (bt of 85); a group of two reference western small-footed bats (ASNHC12082WSB and F54054WSB) together with one sample (15AB0782wsb) from this study (bt of 58); a large group of samples including 6 reference sequences that represent the California bat (bt of 68). Within the *M*. *lucifugus* complex, one large clade of bats from across Canada, but mostly from the eastern provinces (bt of 99), and a smaller clade of bats recovered exclusively from BC (bt of 99), were identified as representing the little brown bat. These assignments were well supported by comparison with sequences in BOLD. The remaining members of this complex formed a well-supported clade (bt of 84), the majority of which were identified as Western long-eared bats based upon morphological re-examination of some of the specimens, as well as the known range of this species. Within this group, two distinct clusters representing other species were identified. The Keen’s bat, which has a highly restricted range in the coastal region of British Columbia, was represented by a group of specimens originating from this area and that clustered closely with a vouchered specimen (UAM23338KEB) identified to this species (bt of 98); a single sample from Washington state, which clearly clustered with this group but was identified as a western long-eared bat (WA2020LEB), may be misidentified since the Keen’s bat range is known to extend to part of that state. Notably, all the test specimens within this clade had originally been identified as the western long-eared bat. Another clade of six bats, which received only modest support (bt of 54), comprised all four reference sequences for the fringed bat as well as two reference specimens of the western long-eared bat, thereby indicating the challenges posed in differentiating these species; three of the reference fringed bats which formed a strongly supported group (bt of 99) may truly represent this species. Based on the relative rarity of the fringed bat in British Columbia, where its range is restricted to the dry interior, the large cluster of genetically similar specimens located within the FRB/LEB clade most likely represented the western long-eared bat.

Barcoding supported changes to the species assignment for 56 of 149 bats examined by this method compared to their original designations determined using morphological keys (S1 Table).A number of these specimens would be misidentified to species when assessed by morphological examination only. Within the *Myotis* genus, the most common misidentifications involved incorrect assignment of specimens as the little brown bat; misidentification was most pronounced for bats of British Columbia because of the much greater species variety encountered in this province.

### RABV phylogeny

**Fig 2:**
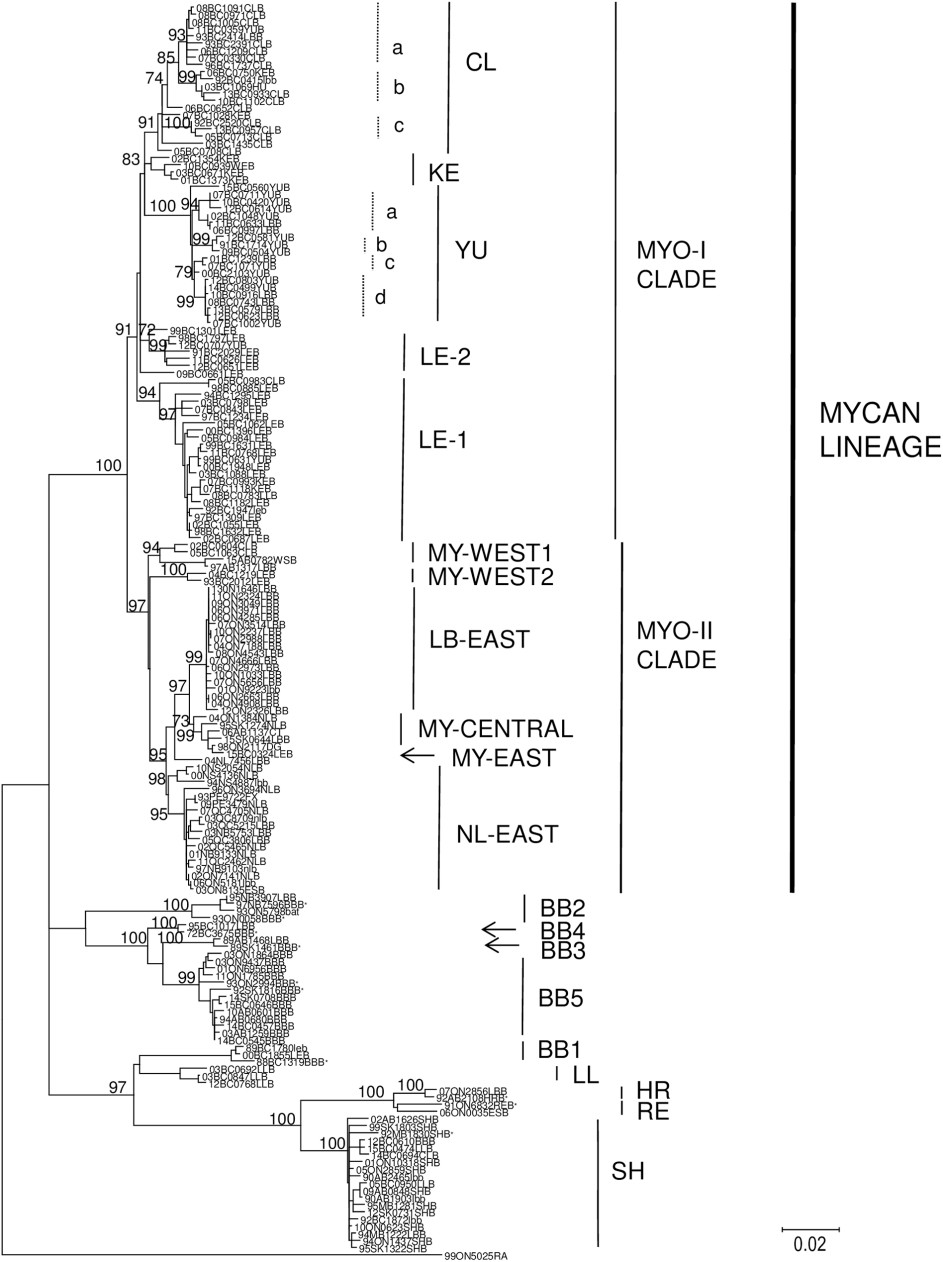
A ML tree identifying multiple rabies virus variants associated with *Myotis* bats. This ML tree was generated using 175 RABV N gene sequences. The dataset includes 164 isolates (see S1 Table) together with 10 selected reference sequences (identified under the CAN-MYO dataset in S3 Table) which are marked by * and a raccoon RABV isolate used as an outgroup. The host species from which each isolate originated is indicated either in lowercase (based on bat specimen morphology) or uppercase (confirmed by barcoding). Bt values > 70 for major internal nodes are shown. The variant, clade and lineage designations are indicated to the right of the tree.

**Fig 3:**
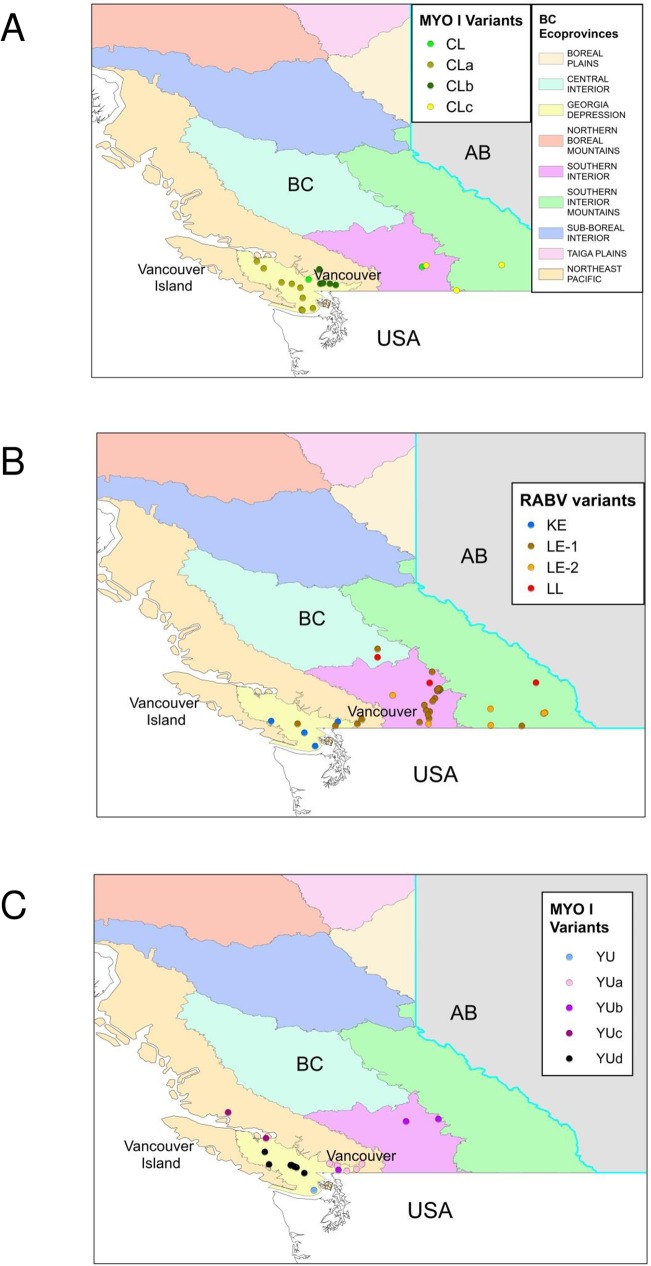
Maps of British Columbia showing the distribution of RABV variants. Maps illustrating sub-division of the province into several ecosystems or ecoprovinces show the locations from which MYO-I and LL RABV variants were recovered thus: (A), all CL subtypes; (B), LE1, LE2, KE and LL variants; (C), all YU sub-types.

**Fig 4:**
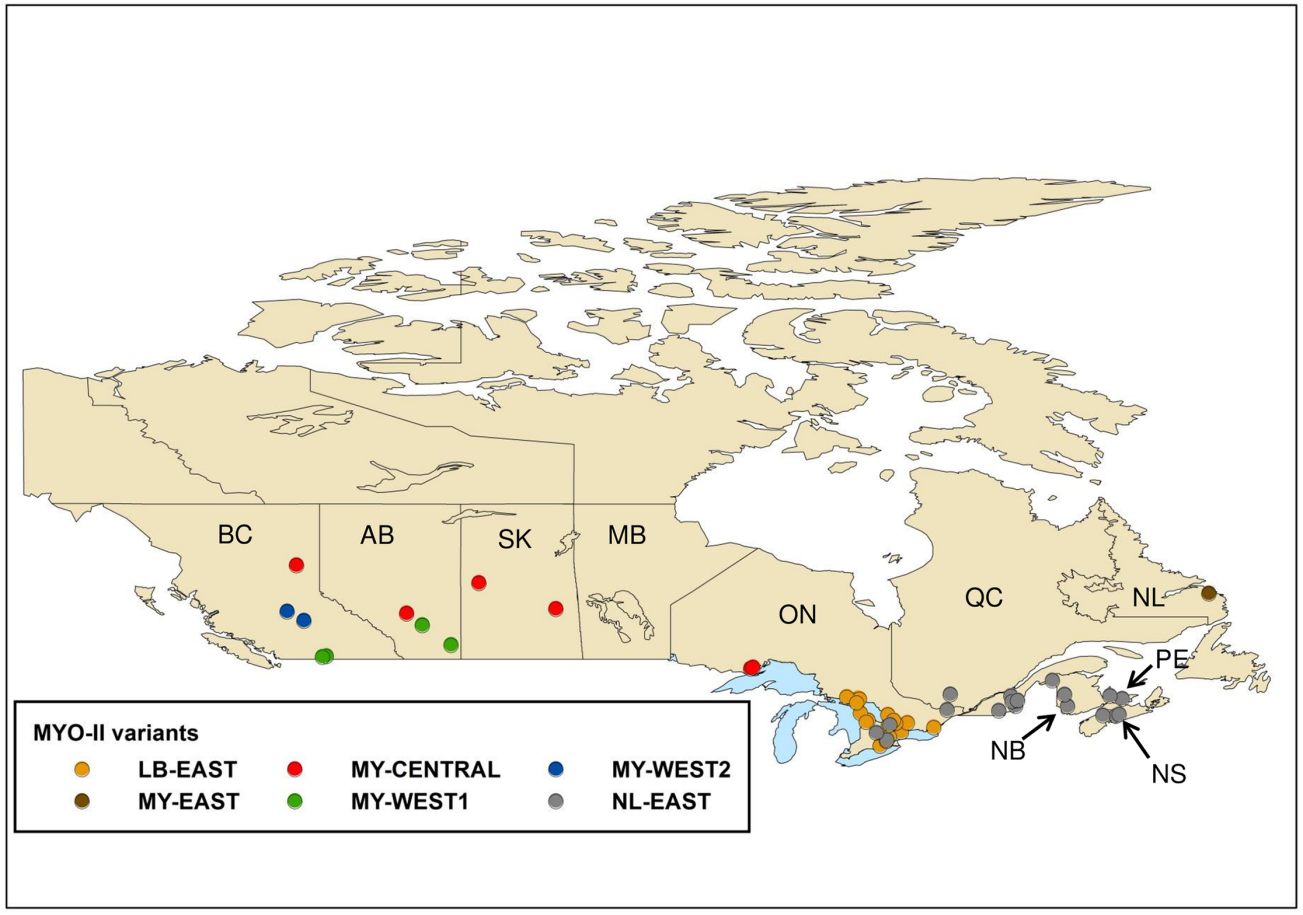
Map of Canada showing the locations of RABV variants. The map illustrates the locations from which all MYO-II RABV variants were recovered.

A ML phylogenetic analysis was performed using the RABV N gene sequences associated with 160 bat specimens (Fig 2), together with 10 reference sequences (see CAN non-*Myotis* dataset, S3 Table), representative of all bat-associated RABV previously known to circulate in Canada, as well as four other isolates (one sample each from fox, dog, cat and human) previously shown to cluster with viruses of the MYCAN type. The majority (124) of these samples clustered within a single large clade designated as the MYCAN lineage (bt of 100), which bifurcates into two distinct clades, MYO-I (bt of 91) and MYO-II (bt of 97). These two clades are in turn further divided into several well supported groups, each of which is designated according to its presumed bat host where possible or according to its geographical distribution as summarised in Table 1. The 75 specimens representing the MYO-I clade, which is restricted to western Canada, and specifically the province of British Columbia, is sub-divided into five groups, LE-1, LE-2, KE, CL and YU; bootstrap values for these clades were 94, 72, 83, 91 and 100 respectively. The 49 specimens representing the MYO-II clade were recovered from across the country, although most samples originated from eastern Canada. MYO-II is subdivided into two large groups, LB-EAST (bt of 99) and NL-EAST (bt of 69), and three smaller groups, MY-CENTRAL (bt of 73), MY-WEST1 (bt of 94) and MY-WEST2 (bt of 100) while a distinct branch, MY-EAST, is defined by a single outlying sample (04NL7456LBB) recovered from the east coast of Labrador. The maps shown in Figs 3 and 4 illustrate the geographical distribution of these RABV variants across Canada.

The greatest richness of variants is distributed in southern British Columbia. The KE variant was recovered from a relatively restricted area comprising the eastern and southern areas of Vancouver Island and the southwestern corner of the province surrounding the city of Vancouver. The YU and CL variants exhibited a more extensive range being distributed further northwards along the coast and into the southeastern British Columbia interior. Representatives of the LE-1 variant came exclusively from the British Columbia mainland, mostly from the southern interior area and extending into the central interior ecological zone, except for one isolate, identified in a long-legged bat that came from the eastern side of Vancouver Island. The LE-2 variant was recovered across the southern interior region of British Columbia and extending into the mountainous zone in the south-eastern corner of the province. Further geographical localisations were evident for certain clusters of isolates within these groupings. For example, within the CL group three sub-groups, CLa, CLb and CLc, were strongly supported with bt values of 93, 99 and 100, while the YU group was subdivided into four sub-groups, YUa, YUb, YUc and YUd, with bt values of 94, 99, 79 and 99 respectively; the potential significance of these subdivisions is described more fully in the discussion. The viral groups comprising the MYO-II clade also exhibit some regional localisation. The NL-EAST variant is found exclusively throughout the Maritime provinces of eastern Canada and in southern Quebec with a few specimens coming from southern Ontario. There appeared to be a clear dichotomy between samples from the province of Nova Scotia (bt of 98) and the remaining specimens of this group (bt of 95). The LB-EAST variant is the predominant type across southern Ontario. A group of six samples recovered from across a geographically wide area from western Ontario, through the western provinces of Alberta and Saskatchewan and into British Columbia, formed the distinct MY-CENTRAL group. The two small MY-WEST groups form outliers within the MYO-II clade. Four samples, two from Alberta and two from the British Columbia interior, three of which were retrieved from communities located close to the Canada-United States border, formed the MY-WEST1 group. Barcoding of the bat hosts for this variant identified one LBB, two CLBs and one WSB, with no identification of the likely host reservoir for this RABV variant. Two MY-WEST2 samples, both isolated from LEB specimens, were recovered from more northern communities in the British Columbia interior.

The phylogeny presented in Fig 2 also identifies several viruses that cluster outside of the MYCAN lineage. A total of 18 isolates typed as the silver-haired (SH) bat variant (bt of 100); this included all 10 specimens which barcoded as silver-haired bats, four little brown bats, of which only one was confirmed to species (94MB1222LBB) and two confirmed long-legged bat specimens (05BC0950LLB and 15BC0474LLB). One specimen each of a big brown bat (12BC0610BBB) and a California bat (14BC0694CLB) were also infected with the SH type. Two spill-over events involving viruses normally associated with lasiurine bats were identified (bt of 100); specimen 06ON0035ESB was infected with a red bat viral variant while 07ON2856LBB was infected with a hoary bat variant. A number of specimens harboured viral variants associated with big brown bats: all 11 submissions that barcoded as big brown bats contained the BB5 variant (bt of 99), two isolates (95NB3907LBB and the unidentified bat 93ON5798bat) were infected with the BB2 variant (bt of 100), 89AB1468LBB harboured a BB3 variant (bt of 100), and 95BC1017LBB was infected with a BB4 variant (bt of 100).

**Fig 5:**
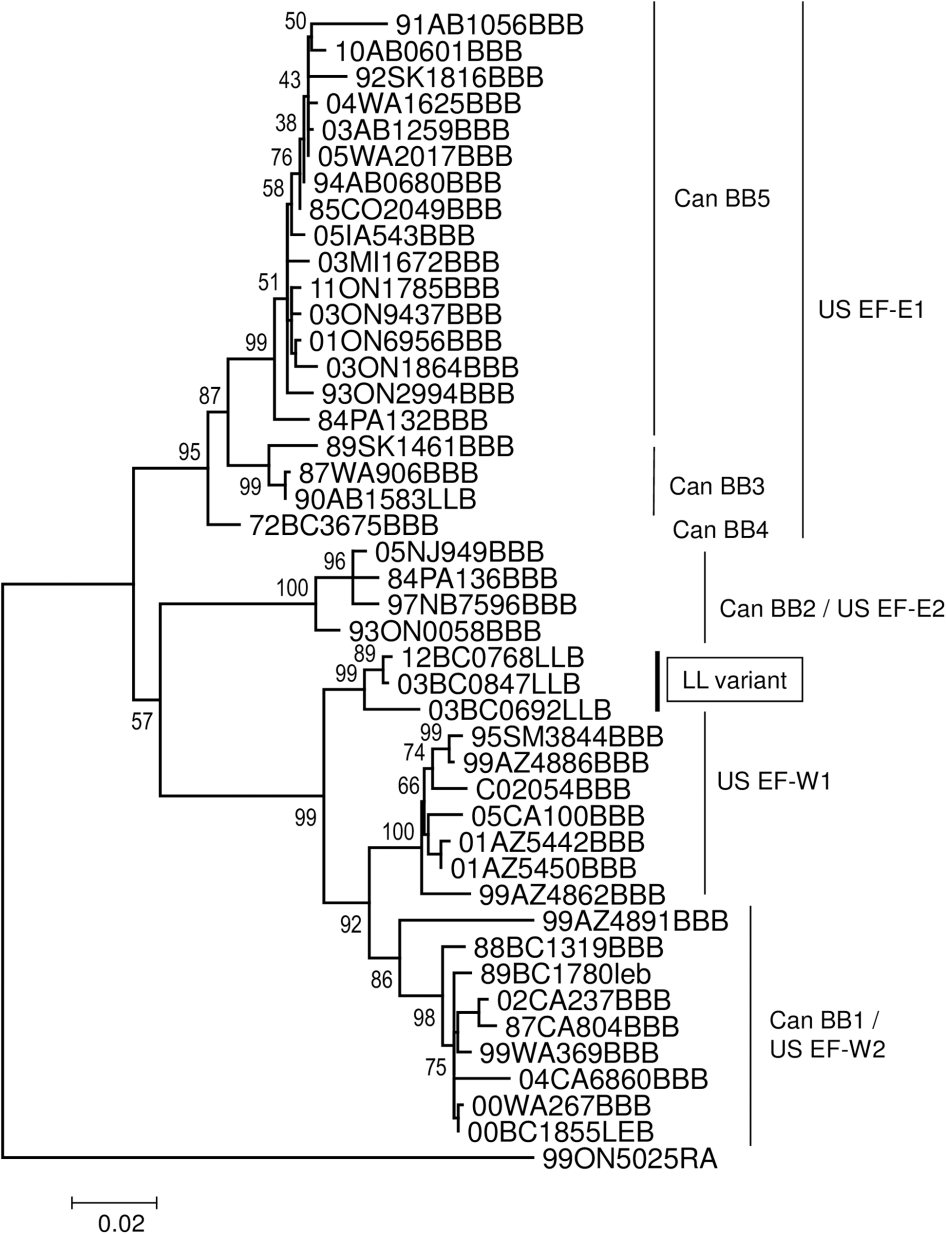
Phylogenetic relationship of five *Myotis*-associated RABVs with viruses of the big brown bat. This ML tree was generated using partial RABV N gene sequences analysed with the Tamura-3-parameter +G+I model of nucleotide substitution with 500 bootstrap replicates. Bt values are shown at all internal nodes and viral variant designations are identified to the right of the tree according to both the Canadian and United States typing schemes.

A more complicated situation was found within the clade representing the BB1 variant (bt of 71). Two isolates, 89BC1780leb and 00BC1855LEB harboured viruses that clustered closely but on a different branch to the BB1 reference sequence (bt of 100). Three closely related viruses (bt of 100), apparently representative of a much more divergent outlying variant, designated here as LL, were associated with long-legged bat specimens (03BC0692LLB, 03BC0847LLB and 12BC0768LLB) recovered from central and eastern areas of the southern British Columbia interior including one recovered close to the Alberta-British Columbia border (Fig 3). The relationship of these five viruses to the BBI variant was further explored by a phylogenetic comparison (Fig 5) with a set of 40 sequences representative of all known big brown bat variants across the United States and Canada (BB dataset, S3 Table). Since different RABV nomenclature for these viral variants has been developed in the two countries, the clades identified in this tree are identified by both systems; their equivalence has been documented previously [27]. By this analysis the viruses from specimens 89BC1780leb and 00BC1855LEB cluster separately within the Canadian BB1 clade (bt of 98) and appear to represent spillover infections from the big brown bat reservoir. In contrast, the viruses from the three long-legged bats (LL variant) cluster as a group (bt of 99) and form an outlying branch to both US EF-W1 and US EF-W2 (Can BB1) clades and clearly represent a distinct viral lineage (Fig 5). One additional viral sample from a long-legged bat (90AB1583LLB) that was not included in the original phylogeny due to availability of limited N gene sequence was also included; it clustered within the BB3 clade (bt of 99) and also appears to represent a spill-over infection.

### Context of *Myotis*-associated rabies viruses throughout the Americas

To further explore the epidemiological relationship of the Canadian *Myotis-*associated RABV lineage within a broader continental perspective, additional phylogenetic analysis was conducted using partial N gene sequences from a wide range of bat-associated viruses recovered from the NCBI database. To accommodate sequences from as broad a range of isolates as possible, datasets of various lengths were used for phylogenetic analysis and the information gained from use of two of these datasets is described. Inclusion of short sequences (<400bp) expanded the geographical and host species coverage but compromised the robustness of the trees that could be generated.

**Fig 6:**
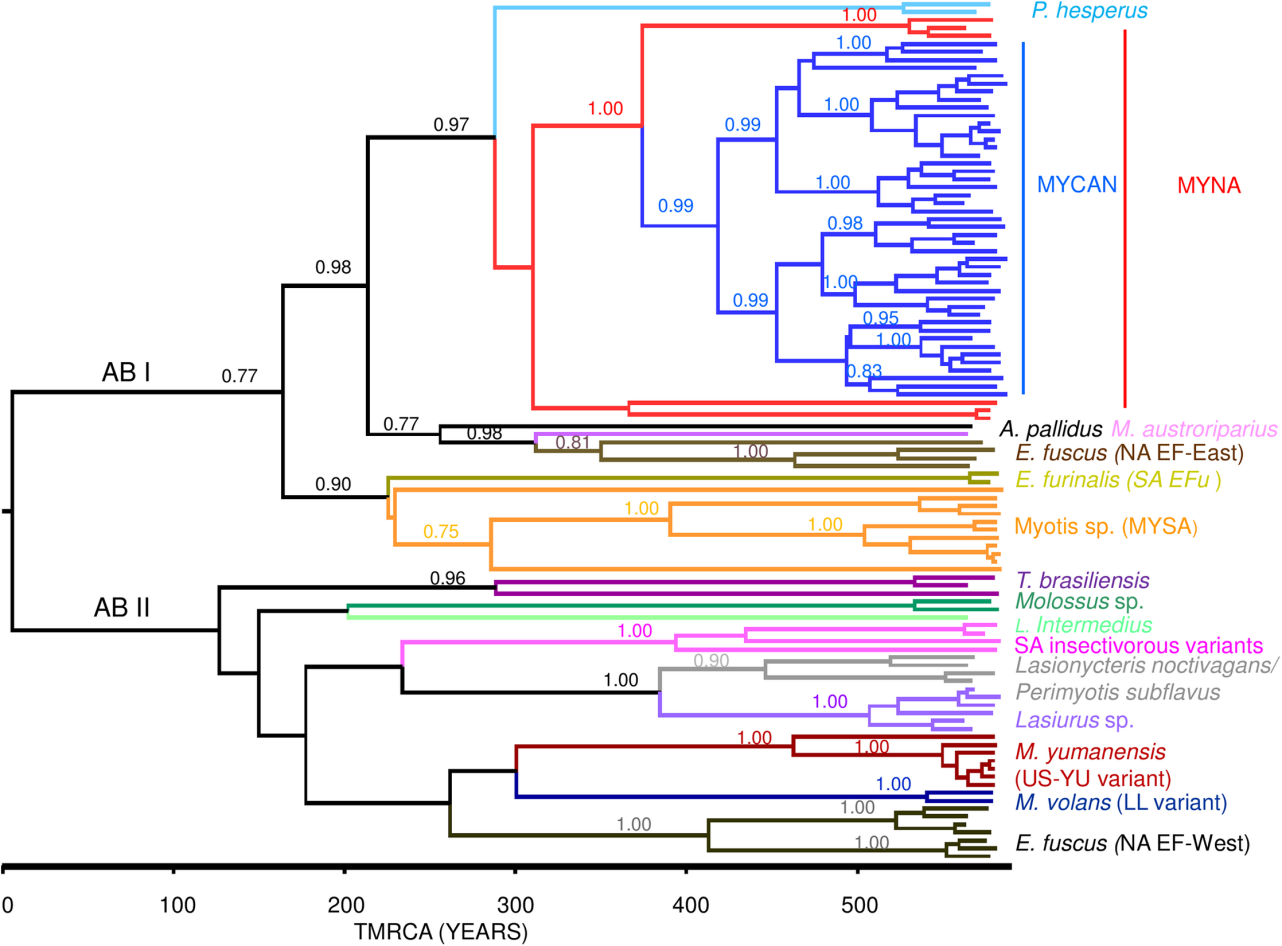
A time-scaled tree illustrating the phylogeny of bat-associated RABVs across the Americas. This mcc tree was generated using the RAMP1 RABV partial N gene dataset which consisted of 114 sequences of 582 bp length. Posterior values are indicated for all major nodes and a time scale in years is depicted below the tree. All main RABV groups are identified to the right of the tree.

Fig 6 presents the mcc tree generated using a dataset (RAMP1) of 114 partial N gene sequences (582 bp) comprising 30 representative MYCAN samples, two of the LL variant, 40 additional sequences of *Myotis*-associated RABVs and 42 sequences representative of the viral variants associated with other insectivorous bats of the Americas (see S3 Table). This tree identifies two major lineages, designated as American bat (AB) I and II, for which the time of the most recent common ancestor (TMRCA) is estimated at 581 years ago. The AB I lineage consists of: the *P*. *hesperus* variant; a North American *Myotis* (MYNA) clade that includes the MYCAN lineage and several isolates from the United States as described below; a variant represented by a single pallid bat isolate (PLB); the eastern North American *Eptesicus fuscus*-associated (NA EF-East) variants together with a single outlying *M*. *austroriparius* isolate; a large group associated with *Myotis* bats, particularly *M*. *nigricans*, of South America, collectively referred to as MYSA and a variant recovered from *E*. *furinalis* bats of Brazil (SA EFu). AB II includes the following main groups: variants associated with *T*. *brasiliensis* with a major dichotomy between those of North and South America; a group of viruses from *Molossus* species of South America and a distinct outlying branch comprising a single isolate from *L*. *intermedius*; another group (SA insectivorous variant) composed of viruses associated with South American *Histiotus* and *Nyctinomops* species; a group of viruses recovered from silver-haired and tri-coloured bats; a clade of *Lasiurus* isolates from North and South America; the US-YU variant that included a distinctive outlier from a *M*. *velifer* bat from Arizona; the LL variant; the western North American *Eptesicus fuscus*-associated (NA EF-West) variants.

A ML phylogeny was generated using a second dataset (RAMP2) that included an additional 54 isolates but covered a sequence length of just 234 bp (S1 Fig). While many internal nodes of this tree exhibited very low bootstrap values, some informative associations were evident. In particular the LL variant specimens (03BC0692LLB and 03BC0847LLB) clustered with strong support (bt of 96) with two *Myotis* samples from Colorado (comsp2057 and comsp5264) while one additional *Myotis* specimen from Colorado (comsp2057) and one from a *M*. *velifer* specimen of Texas (96TX4258mv) clustered as outliers to this clade. Two closely-related *Myotis-*associated specimens from Arizona (95AZ335MYO and 99az488MYO) segregated on a separate outlying branch of the NA EF-West (BB1) clade.

Taken together, analysis of the RAMP1 and RAMP2 datasets identified several United States specimens that clustered within the MYCAN lineage (Fig 6, S1 Fig). These included two viruses from California bats recovered in Washington state that grouped within LE1; a Keen’s bat virus from Washington state that clustered within LE2; one LEB specimen from Washington state grouped within the CLc cluster; one Californian sample (YUB species) grouped within the YU clade identified in British Columbia; four specimens from Idaho and Colorado states were included in the MY-WEST1 group; a total of eight Californian specimens formed a distinct outlying branch (MY-WEST3) associated with the MYO-II clade; one sample from Michigan state recovered from a little brown bat that clustered within the LB-EAST group. Other United States samples that clustered closely with but outside of the MYCAN lineage defined a number of additional variants. Two distinct branches (US-MY1 and US-MY2) were defined by small numbers of samples; two closely-related Florida samples from *M*. *austroriparius* formed a third outlying branch (US-AUS1) while another virus from *M*. *austroriparius* lay on a distinct branch (USAUS2) that appeared to be more closely related to the pallid bat specimen. Collectively, these variants, together with the MYCAN lineage, are referred to here as the *Myotis* North American (MYNA) clade. Several viruses identified as originating from *Myotis* bat species in Colorado (three) and Arizona (four) clustered with big brown bat variants while four Arizona samples and three California samples clustered closely with the variants believed to be harboured by *P*. *hesperus*. These viruses may either represent spill-over infections from their reservoir hosts or instances of incorrect host species assignment.

## Discussion

### Importance of knowledge of bat rabies to public and veterinary health

The perpetuation of rabies in bat populations has serious consequences for public and animal health due to the potential for spill-over infections. In Latin America, due to successful campaigns to control dog rabies, the majority of reported rabies cases in both domestic animals and humans are now due to exposure to wildlife rabies, especially to the variants that circulate in the vampire bat, *Desmodus rotundus* [28,29]. In the United States and Canada, though human rabies is rare, a high proportion of indigenously acquired cases are the result of infection by bat-associated variants; indeed, of the last five human rabies cases reported in Canada between 1985 to the present, four were the result of transmission of bat-associated RABVs [30]. Moreover such spill-over infections have the potential to establish new viral- host associations in which the virus is maintained and transmitted within its new environment as documented by the emergence of skunk rabies in Arizona following a host jump from a bat reservoir [27]. Such events threaten to undermine expensive campaigns to control rabies in other mammalian hosts. Thus, knowledge of the role of particular bat species as disease reservoirs and the distribution of rabies virus variants across the continent provides information on disease risk for humans and animals. Moreover, this knowledge can help to rapidly identify virus host jump events into other mammals that require timely intervention to prevent establishment of new foci of disease.

### Lack of samples limits study of rabies in the *Myotis* genus

This study has overcome two main challenges to a better understanding of the importance of *Myotis* bats as rabies reservoirs. First, despite the frequency with which specimens of this genus are submitted for rabies diagnosis, only small numbers are found to be rabid. Clearly, the prevalence of rabies in *Myotis* bats is very low despite the tendency of passive surveillance methods to be biased towards collection of sick bats with human contact. Consequently, studies of bat rabies in the Americas have usually included only small numbers of bats of this genus and this has confounded efforts to study the role individual *Myotis* species play in RABV maintenance. The 127 cases identified in this report, collected in Canada over 25 years, represent by far the largest study of *Myotis*-associated RABV variants ever undertaken in this country.

### Value and challenges in the application of genetic barcoding

The other challenge addressed in this study is the identification of bat specimens to species, particularly for rabies diagnostic submissions, since these specimens are often physically damaged upon collection and are sometimes incomplete (eg. head only). While genetic methods of species identification, especially those targeting genes of mitochondrial origin, have gained significant following in recent years [19], challenges to correct species assignment persist. A study of bat-associated rabies viruses in the United States [11] in which a heterogeneous group of *Myotis*-associated viruses was identified did not clearly attribute these variants to particular species of this genus. Instead, the authors refer to two groups of *Myotis* species, the *M*. *lucifugus* complex, comprising *M*. *lucifugus*, *M*. *keenii*, *M*. *evotis* and *M*. *thysanodes* and the *M*. *californicus* complex that includes this species as well as *M*. *ciliolabrum* and *M*. *lebeii*. Indeed, in this study use of the BOLD for identification of species within the *M*. *lucifugus* complex was problematic and probably indicative of several challenges including: submission to the database of barcodes incorrectly assigned to species, recent recognition that the taxonomy of North American *Myotis* bats may be flawed by either over or under splitting of the genus or that the mitochondrial genome exhibits incomplete lineage sorting, thereby confounding use of this approach for some members of the genus. Certain species within the *Myotis* genus are paraphyletic by COI barcoding and their accurate discrimination is problematic [31,32]. In Canada, species assignment is facilitated by the smaller number of native *Myotis* species, many of which have quite restricted ranges [15,33]. Two samples originating from a restricted area of southwestern Ontario, that were originally identified as little brown bats, yielded barcodes that clustered within the *M*. *californicus* complex, identifying them as the eastern small-footed bat given that other members of this complex are restricted in their range to western Canada. By a similar process, specimens within the *M*. *lucifugus* complex were assigned to particular species consistent with their biogeography and predicted phylogeny [31]. Most of the specimens in the LEB/FRB clade were assigned as western long-eared bats based on their source locations, similar phylogeny and the rarity of the fringed bat in Canada. Finally, where possible, the remaining carcasses of these specimens were re-evaluated independently by an expert in bat taxonomy to confirm their species assignments. While every effort was made to accurately assign a species to each submission and attribute RABV variants to host species accordingly, future developments in *Myotis* taxonomy may impact some of the conclusions drawn here about the role of certain reservoir species.

The high level of species misidentification (56 of 149) when comparing specimens by barcoding versus morphological traits was due primarily to challenges in differentiating between members of the *Myotis* genus, but in addition 15 bats (10 big brown bats and five silver-haired bats) were erroneously identified as little brown bats. The latter observation brings into question a previous conclusion [17] that the little brown bat was a frequent victim of spill-over infections from the silver-haired bat. Moreover, during compilation of datasets of RABV sequences deposited into the public databases there were many instances in which viral sequences believed to have originated from *Myotis* bats clustered within clades representative of variants of other bat species [34,35]. These instances may represent rabies virus spillover into *Myotis* bats or be the result of host species misidentification. Such findings reinforce the need for improved species identification to reliably infer the RABV reservoir role of many bat species in the Americas and routine genetic characterisation of rabid bat submissions targeting the COI gene or potentially other loci would significantly improve knowledge in this area.

### Relationship between distribution of MYCAN lineage variants and bat host ecology

This study has shown that a single progenitor RABV was responsible for the emergence of the majority of viruses (MYNA group) that now circulate in *Myotis* species across North America. The MYCAN lineage that evolved from the MYNA group can be differentiated further into several distinct variants that currently circulate regionally in parts of Canada and the UnitedStates over ranges that, to some extent, reflect the geographical distribution of their respective host species.

Both the little brown bat and the northern long-eared bat range across most of the southern areas of all 10 Canadian provinces [15] but they appear to be prominent RABV hosts only in eastern Canada. The little brown bat harbours the LB-EAST variant distributed throughout south-eastern Ontario and into parts of the United States, based on a single isolate from Michigan. A single distinctive isolate (MY-EAST) was recovered from a little brown bat on the east coast of Labrador, which may suggest a broader circulation of variants associated with this species. The northern long-eared bat harbours the NL-EAST variant throughout the eastern maritime provinces, Quebec and some areas of southern Ontario and this species may also be the reservoir for the MY-CENTRAL variant, recovered over a large area from western Ontario to Alberta. However, given the small number of isolates of this variant, including two from non-bat species, confirmation of the host reservoir for this variant will require additional isolations.

Despite their extensive overlap in range, little brown and northern long-eared bats do differ in their foraging and roosting preferences [15]. Northern long-eared bats prefer to forage on forested hillsides and their roost sizes are relatively small (<100 individuals) compared to little brown bats which have been known to form nursery colonies of many hundreds of individuals. Little brown bats frequently forage over water or sometimes around trees on relatively open areas. Consequently, although spill-over events are certainly possible (the NL-EAST variant was confirmed in three little brown bats and one eastern small-footed bat), ecological factors may tend to restrict these two viral variants to their specific hosts.

The reservoir species for the outlying branches of the MYO-II clade (MYWEST1, MY-WEST2 and MY-WEST3) are less clear due to limited numbers of specimens in each of these clades, but species restricted to western Canada may be important. In both Canada and the United States, the California bat was the host for three of five isolates of MY-WEST1, and for four of seven isolates of MY-WEST3 in the state of California, while the western long-eared bat was the host for both MY-WEST2 isolates.

The situation in western Canada, and specifically in British Columbia, is complicated, because of the province’s highly diverse climate and physiography, which results in a much greater diversity of native bat species. British Columbia is divided into ten ecologically distinct areas or ecoprovinces ranging from wet forested pacific coastal regions and mountainous terrain to the arid interior plateau of the Okanagan valley and northern boreal regions. These diverse foraging and roosting opportunities support nine native *Myotis* species [33]. However, this study suggests that only four of those species, the western long-eared, California, Yuma and Keen’s bats are important RABV reservoirs in the province. Of these, the western long-eared bat appears to act as the reservoir for two distinct clades of viruses (LE-1, LE-2) that exhibit distinct but overlapping geographical distributions. This species is reported to occur at relatively high elevations compared to most other bats and is thus distributed widely across much of the province. Its range extends into southern parts of Saskatchewan and Alberta and across much of the western United States.

The California bat, which harbours the CL viral clade, also ranges over several parts of the western United States, but in Canada it is restricted to the southern parts of the British Columbia mainland and most of the pacific coastal region. The apparent emergence of several distinct local CL RABV variants may reflect the distribution of two sub-species of this bat as detailed by Nagorsen and Brigham [33]. *M*.*c*. *caurinus* ranges throughout coastal regions of southern Alaska and British Columbia, in areas where the CLa viral subtype was prominent; indeed, the 9 specimens of CLa (bt of 93) originated exclusively from Vancouver Island. In contrast, *M*. *c*. *californicus* inhabits the mainland interior, including the southeastern corner of mainland British Columbia, where the three specimens of the CLc subtype (bt of 100) originated. These viral variants may have evolved as a result of limited contact between these sub-species. The five CLb variant specimens (bt of 99) were recovered exclusively from the southwestern part of the mainland, and this variant was responsible for the only human case, sample 03N1069HU, [36] to be included in this study. While human rabies cases due to bat-associated RABVs are rare throughout North America, a relatively high proportion is due to the silver-haired bat RABV variant [18]. Spill-over of *Myotis*–associated RABVs to humans has on occasion been reported in the United States [37,38], but unfortunately no detailed information on the nature of the viruses involved in these cases is available for comparison.

The Yuma bat has a range in British Columbia rather similar to that of the California bat and the YU variant viruses associated with this species could also be sub-divided according to geographical location. The YUa (six specimens, bt of 94) and YUb (three specimens, bt of 99) clades comprise samples recovered from different parts of the British Columbia mainland; YUa was recovered from a region east of Vancouver while two of the YUb samples were from the southern interior of the province. The YUc subtype (3 specimens, bt of 79) comprised samples from both the mainland and island areas of the coastal (Northeast Pacific) region and the YUd subgroup (7 specimens, bt of 99), comprised of six samples from Vancouver Island and one from a close neighbouring island together with the group outlier (15BC0560YUB) also from Vancouver Island are distributed throughout the region referred to as the Georgia Depression. Seven specimens infected with the YU variant were identified as little brown bats and the role of this latter species as a reservoir for some of these variants could be further investigated with future additional isolates. The YU variant identified in British Columbia and in one isolate from California is quite distinct from the variant previously identified in Yuma bats in other parts of the US (YU-US) and indicates independent emergence of RABV lineages in this species on at least two occasions.

The Keen’s bat, which has a very restricted range in the Pacific coastal forest region and is known to range primarily in British Columbia and parts of Washington state [33], is proposed as the reservoir for another RABV variant (KE) represented by just four isolates, one from a Western big-eared bat. Given the limited population size and range of this species, identification of a viral variant that appeared to associate specifically with it was surprising.

All of the rabies cases identified for inclusion in this study originated from southern areas of the country and did not fully respect the complete known range of many of these bat species. This was certainly the case in BC where despite the recorded range of many *Myotis* species into the central regions of the province, rabid specimens are recorded from southern areas only. It is unknown if this is an artifact of passive surveillance, due to limited submissions from more northern areas where human populations are relatively sparse, or if it reflects the true range of these viral variants in these bat populations. Modelling studies suggest that maintenance of RABV in temperate zone bat populations relies upon a complex dynamic involving virus incubation times, host hibernation and the annual bat birth pulse [39]. At the limits of a bat species range these dynamics may be shifted such that the virus may be more prone to fade-out events that prevent its maintenance over the entire host range.

### A continental perspective on the importance of *Myotis* bats as rabies hosts

The age of the American bat RABV lineage has been estimated previously using different segments of the genome. Using Bayesian methods applied to complete N gene sequences, Hughes et al. [40] estimated the age of this lineage at 357 years (corresponding to the TMRCA of 1660, 95% HPD 1267–1782), while Kuzmina et al. [41], using a G gene sequence dataset, reported an earlier TMRCA of 732 years (95% HPD 436–1107 years). This study used partial N gene sequences to estimate the TMRCA of this lineage at 581 years (Fig 6), corresponding to year 1434 (95% HPD 586–1773), a value in line with those previous reports, given the wide ranges associated with these estimates. Notably, the phylogenies described in this report (Fig 6, S1 Fig) clearly indicate that during the evolution of this lineage multiple host jumps of viruses from other bat species into members of the *Myotis* genus have occurred and, in some cases, have resulted in long term perpetuation in these new hosts. Consistent with previous observations [6], this study identifies two major clusters of *Myotis*-associated RABVs in North and South America.

The two main reservoir species of the South American RABV variants, *M*. *chiloensis* and *M*. *nigricans*, belong to the neotropical *Myotis* subclade and are phylogenetically distinct from most members of the nearctic subclade that inhabit North America. The geographical separation of these two groups of *Myotis* bats explains the independent introduction and circulation of distinct RABV variants in these two populations. The spill-over of RABV variants from the *Lasiurus* and *Eptesicus* genera and from silver haired bats into *Myotis* bats observed in this study is consistent with previous suggestions that these reservoirs form important sources of RABV transmission into *Myotis* bats and that on occasion such events can result in fixation of new virus-host associations [42,43]. Indeed the LL variant identified in this study appears to have a common origin with the western big brown bat virus group although the observation that this variant appears to circulate in other *Myotis* species south of the Canadian border (Fig 6, S1 Fig) raises questions as to the true identity of the host of this variant and its range.

In addition to spill-over of RABV variants from non-*Myotis* bats, this study identified a number of instances in which a MYCAN variant was found in another *Myotis* species other than the one presumed to represent the true reservoir species. The dataset produced by these studies provides an excellent opportunity to examine the factors that may impact spill-over infections between closely related species.

Furthermore, this dataset included spill-over infections into three mammalian carnivores. Two of these spillover events involved domestic animals and underline the public health implications of rabies in bat reservoirs. The third spillover case involved a fox from PEI, representative of three rabid foxes reported in this province in 1993, all of which harboured the NL-EAST RABV variant. While these cases raised concerns at the time of possible fox to fox transmission of this virus [44], no cases involving this variant have been reported in this province subsequently.

### Factors impacting bat populations and their future role as rabies reservoirs

The rapid spread of white nose syndrome in eastern North America, first identified in a bat cave hibernaculum in New York State in 2006, has had a devastating impact on populations of certain bat species, in particular the little brown and northern long-eared bats [45]. The reduction in these bat populations could have significant impact on the phylogeography of bat rabies throughout the region. Ongoing surveillance will determine if the resulting extensive decline in these bat populations reduces their levels such that rabies virus transmission can no longer be maintained. Such effects could lead to the elimination of some of the rabies viral variants described here in some areas. Ongoing surveillance will be needed to test such theories and to provide early warnings for any new virus-host associations resulting from RABV transmission, either to *Myotis* species from other sources or from *Myotis* species to other mammals.

## Supporting information

S1 Fig ML analysis of the RAMP2 dataset. This ML phylogenetic tree was generated from partial N gene sequences of bat-associated RABVs using the Tamura-3-parameter +G+I nucleotide substitution model with 500 bootstrap replicates. Many of the branches described in the text are highlighted in red.
(PPT)

S1 Table Listing of the 165 Canadian bat specimens examined in this study. (XLS)

S2 Table Specimens providing reference COI sequences used for interpretation of barcoding data. (XLS)

S3 Table Additional bat-associated RABV isolates included in the phylogenetic analyses. (XLS)
