# Supplementary material for: The phylogeography of Myotis bat-associated rabies viruses across Canada
Source: PLoS Negl Trop Dis. 2017 May 19;11(5):e0005541. doi: 10.1371/journal.pntd.0005541 (PMC5453604; doi:10.1371/journal.pntd.0005541)
Supplement: S1 Fig — This ML phylogenetic tree was generated from partial N gene sequences of bat-associated RABVs using the Tamura-3-parameter +G+I nucleotide substitution model with 500 bootstrap replicates. Many of the branches described in the text are highlighted in red. (PPT) [file pntd.0005541.s001.ppt]

## Slide 1
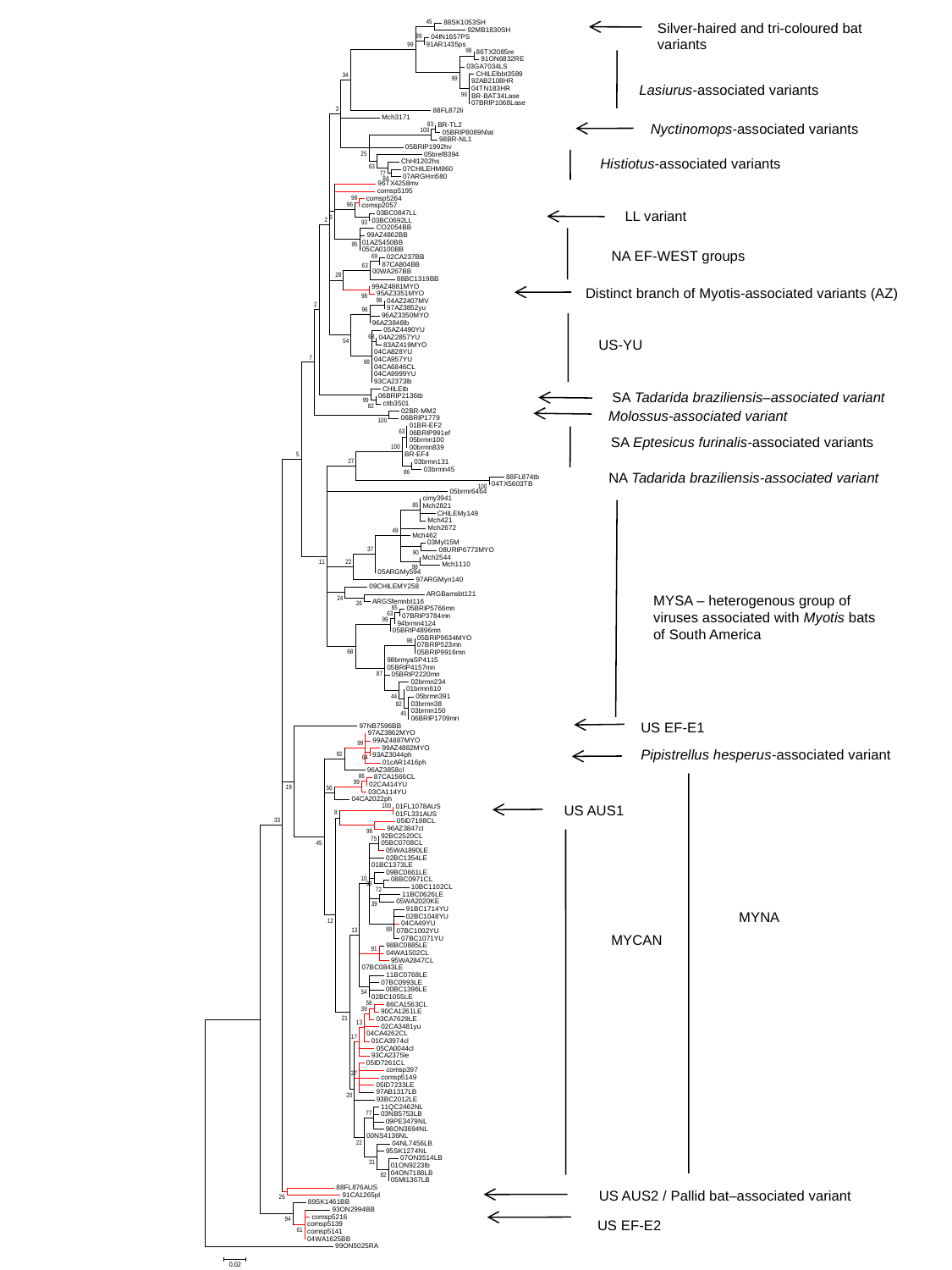

Silver-haired and tri-coloured bat
variants
Lasiurus-associated variants
Nyctinomops-associated variants
Histiotus-associated variants
LL variant
NA EF-WEST groups
Distinct branch of Myotis-associated variants (AZ)
US-YU
SA Tadarida braziliensis–associated variant
Molossus-associated variant
SA Eptesicus furinalis-associated variants
NA Tadarida braziliensis-associated variant
MYSA – heterogenous group of viruses associated with Myotis bats of South America
US EF-E1
Pipistrellus hesperus-associated variant
US AUS1
MYNA
MYCAN
US AUS2 / Pallid bat–associated variant
US EF-E2
